# Individual-based and interactional resilience mechanisms in social and healthcare service NPOs during the COVID-19 pandemic: Handling a disruptive extreme context in Austria

**DOI:** 10.3389/fpsyg.2022.897790

**Published:** 2022-08-09

**Authors:** Katharina Anna Kaltenbrunner, Sandra Stötzer, Birgit Grüb, Sebastian Martin

**Affiliations:** ^1^Department of Strategic Management & Organization, Paris Lodron University of Salzburg, Salzburg, Austria; ^2^Department of Public and Nonprofit Management, Johannes Kepler University of Linz, Linz, Austria; ^3^Department of Management Accounting, Johannes Kepler University of Linz, Linz, Austria; ^4^Department of Health, Social and Public Management, University of Applied Sciences Upper Austria, Linz, Austria

**Keywords:** resilience, COVID-19 pandemic, social and healthcare service NPOs, individual mechanisms, interactional antecedents

## Abstract

While Austrian social and healthcare service nonprofit organizations (NPOs) are key performers in the COVID-19 pandemic, we also notice their vulnerability in terms of struggling with this disruptive extreme context. The particularity of disruptive extreme contexts is that organizations commonly can neither anticipate them, nor prepare specific countermeasures or specialized resources for fighting against them. Thus, we regard organizational resilience based on non-specialized resources as an appropriate approach for dealing with (the struggles of) disruptive extreme contexts. Organizational resilience refers to an organization's ability to resist disruptive extreme contexts while maintaining and adapting functionality and ultimately learning from these extreme contexts by mobilizing and accessing the required resources, behaviors and capabilities. Based on 33 expert interviews with NPO top and middle managers we aim to explore individual-based and interactional resilience mechanisms of NPOs in the pandemic. The qualitative content analysis yielded to following results: Individual personality traits (e.g., pragmatisms, flexibility) and attitudes (serenity and optimism) constitute individual-based resilience mechanisms. Moreover, a shared (crisis) understanding (e.g., common sense of direction), social connectedness (e.g., team cohesion) and managerial staff orientation (e.g., a caring attitude) as interactional resilience mechanisms helped to maintain and adapt NPOs' functioning. Overall, this study reinforces the multilevel nature of resilience in terms of the crucial combination of individual and interactional resilience mechanisms for facing adversity. Moreover, it emphasizes the evolving nature of resilience in terms of the required time for, e.g., building trust.

## Introduction

The abrupt outbreak and ongoing threat of the COVID-19 pandemic have made the need of resilience even more clear (Barton et al., 2020; Guistiniano et al., 2020). The pandemic sent a jolt across the globe and resulted not only in a health, but also in a social and economic crisis (Brammer et al., 2020; Hutton et al., 2021; Kuenzi et al., 2021; Sarkar and Clegg, 2021). It caused a worldwide disruption of business models, global institutional alignments, social and political processes as well as organizational disruptions (Lewin et al., 2020; Sarkar and Clegg, 2021). Thus, it has affected citizens, governments, businesses and nonprofit organizations (NPOs). The pandemic hit NPOs hard by creating financial and organizational challenges (Deitrick et al., 2020). Although the crisis highlighted their vulnerability, many NPOs worldwide also were crucial players in mitigating its devastating effects (Shi et al., 2020; Kim et al., 2022), like in Austria, where NPOs have a decisive role in coping with the pandemic since its beginning in March 2020. This is notably the case for social and healthcare service NPOs that offer multiple care and counseling services. A continuous service supply or (sometimes) even an extension of services was necessary (Meyer et al., 2021; Millner et al., 2021).

We refer to the COVID-19 pandemic as an extreme context, which constitutes an intense, risky, and often dangerous environment (Maynard et al., 2018) or is even life-threatening (Mithani, 2020). Extreme contexts involve constraints, such as time pressure or emotional constraints on rationality, such as fear (Hannah et al., 2009). The pandemic represents the specific occurrence of a disruptive extreme context. Such extreme contexts are the “most extreme punctuation of normalcy” due to their core feature of substantial organizational, economic, political or social disruptions (Brammer et al., 2020). Corresponding negative effects—be they physical, psychological, or material—are unavoidable (Hannah et al., 2009). Moreover, these contexts have a surprising, unforeseen nature. Thus, organizations commonly can neither anticipate them, nor prepare specific countermeasures or specialized resources like emergency plans (Hällgren et al., 2018).

Drawing on the work of Dayson et al. (2021) and Hutton et al. (2021), we propose that providing services during a pandemic requires organizational resilience, which refers to the organizational ability to resist adversities while maintaining and adjusting operations, e.g., in terms of service delivery (Sutcliffe and Vogus, 2003; Van der Vegt et al., 2015; Witmer and Mellinger, 2016; McCarthy et al., 2017). This is due to its “emphasis on prompt and autonomous recovery that does not rely on specialized resources” (Mithani, 2020, p. 509). The fact that resilience is based on non-specialized resources makes it also suitable for coping with disruptive extreme contexts. Non-specialized resources are resources not prepared specifically for a certain disruption (i.e., a specific threat), but rather include general individual resources (e.g., emotion efficacy), relational resources (e.g., sound relations) or organizational ones (e.g., general preparedness).

Resilience derives from the Latin term “resilire”, which means to “jump back” to a former position (Guistiniano et al., 2020). Bouncing back to an earlier “normal” (original equilibrium) refers to static resilience that corresponds with an internal outlook. Systems only focus on internal repairing and reconstructing, which is almost impossible in complex situations. Dynamic resilience, in contrast, assumes that it is not possible to return to the original. It aims at finding an “adjusted optimality”, i.e., a new equilibrium or even new equilibria, as it is the case in the corona pandemic. Thus, dynamic resilience contributes to evolution (Mithani, 2020).

There are different conceptualizations of organizational resilience. Scholars refer to this concept as the ability to withstand adversity or to absorb and recover from shocks, organizational responses to external threats, organizational reliability, the adaptability of business models or design principles for limiting disruptions of supply chains (Linnenluecke, 2017; Duchek, 2020; Hillmann and Guenther, 2021; Jalil et al., 2021). Thus, it can represent a capacity, ability, capability, quality, property or even a process (Hillmann and Guenther, 2021). We follow a (c)apability-based perspective, because it particularly offers insights into the internal workings of resilience and the necessary conditions to further develop it. A capability-based view also has a genuine practical value, as it shows, how practice may attain resilience (Duchek, 2020). In our paper, we understand organizational resilience as an organization's ability to resist disruptive extreme contexts while maintaining and adapting functioning and ultimately learning from these extreme contexts by mobilizing and accessing the required resources, behaviors and capabilities (c.f. Sutcliffe and Vogus, 2003; Van der Vegt et al., 2015; Witmer and Mellinger, 2016; McCarthy et al., 2017; Hillmann and Guenther, 2021).

Organizational resilience has a multilevel nature; it can refer to individuals, teams, organizations, and other systems (like societies). Thus, it reflects individual, team, organizational or societal resilience (Witmer and Mellinger, 2016; Williams et al., 2017; Jalil et al., 2021). The framework by Raetze et al. (2021) integrates individual, team and organizational resilience. They illustrate antecedents, conceptualizations and outcomes of resilience on these three levels and analyze how they are linked. However, there is no consensus regarding the interrelationship of resilience levels. On the one hand, organizational resilience is considered a precursor for, e.g., individual resilience; on the other hand, individual resilience is said to predict organizational resilience. These authors also discuss the antecedents of resilience levels. There are level-specific antecedents (e.g., individual job expertise as an antecedent for individual resilience), but also multilevel antecedents which enable more than one resilience level (e.g., humor is supposed to enhance all three resilience levels).

In accordance with our understanding of organizational resilience, we define resilience mechanisms as non-specialized resources at the individual, relational and organizational level that antecede organizational resilience and thus enable organizations to resist extreme contexts in terms of adapting and maintaining operations. Individuals' stable attributes (e.g., openness to experience), skills and competences (like reflexivity, sense making, creativity, management skills) as well as emotional resources and attitudes (e.g., optimisms, gratitude) can constitute individual-based resilience mechanisms (Hillmann and Guenther, 2021; Raetze et al., 2021). Interactional resilience mechanisms refer to social resources, such as social connections, support, trust, cohesion or network relationships (Williams et al., 2017; Hillmann and Guenther, 2021; Raetze et al., 2021). In order to systematize interactional mechanisms, we point to social capital as general resources embedded in or generated from relations (Nahapiet and Ghoshal, 1998; Adler and Kwon, 2002; Williams et al., 2017). We focus on relational social capital that is created and leveraged through relations and cognitive social capital “which represents shared understanding, interpretations and systems of meanings between parties” (Nahapiet and Ghoshal, 1997, p. 35). The former relates to network capital (e.g., team cohesion, social support), leadership capital (e.g., employee orientation or fairness) and value/beliefs capital (e.g., common beliefs or trust) (Badura et al., 2013).

There is only limited research that investigates, which mechanisms underpin organizational resilience of NPOs in disruptive extreme contexts (Hutton et al., 2021), and even less discussing in-depth, how individual, relational and organizational mechanisms influence their resilience (Herberg and Torgersen, 2021). Our review of current research shows that studies predominantly focus on organizational-level mechanisms and refer to financial, structural, human and social resources, strategies and practices (Raetze et al., 2021). Hutton et al. (2021), for instance, provide an empirically based framework that illustrates the interconnectedness of nonprofit and community resilience in the context of the combined pandemic-hurricane threat in New Orleans. They suggest that NPO resilience draws on mission orientation, strategic planning, resource management, external communication, board leadership, and operational capacity. Another qualitative study by Searing et al. (2021) analyzed human-service providers in the financial crisis caused by the 2015–2017 Illinois Budget Impasse. They consider NPO resilience to consist of five tactical themes (i.e., financial, human resources, outreach, programs and services, as well as management and leadership) and corresponding subordinate resiliency tactics. Besides, Dayson et al. (2021) explored how local community organizations supporting the elderly handle the pandemic. They conceptualize organizational resilience as absorptive, adaptive and transformative capacity. Their qualitative study shows that at first, NPOs focused on how to continue service delivery (through absorptive capacity) while later on they concentrated on how to adapt it. Adaptation involved ongoing adjustments, innovations and several enabling mechanisms including tangible factors (like sufficient resources) and intangible ones (e.g., guiding values or leadership). Finally, Kim et al. (2022) studied the social welfare sector in Texas (US) in the disruptive context of hurricane Harvey. They propose that “hybrid organizing” in terms of combining formal with informal structures enhances resilience capacity. Formal structures form the basis for informal relations or networking. Besides, disruptions from disasters can impair formal relations and provide space for informal ones.

Moreover, there are scholars who apply a multilevel view and explore how individual-based and interactional (as well as organizational) mechanisms influence organizational resilience. Herberg and Torgersen (2021), for instance, studied organizations in Norway and identified six resilience mechanisms applied in unforeseen and uncertain events (e.g., terrorist attacks). These are general preparedness (e.g., plans, training or equipment), characteristics and competence of individuals (e.g., attitudes, emotional competence or mental abilities), sound relations (e.g., organizational culture), creative behavior and improvisational skills, the ability to reflect and learn, and finally emotion efficacy in terms of the ability to handle one's emotions. A second study by Witmer and Mellinger (2016) investigates two US healthcare NPOs who experienced fundamental funding changes. These authors identified six factors characterizing organizational resilience (incl. individual-based and interactional ones): a commitment to the NPO's mission, the ability to improvise using existing resources, reciprocal relations with the community based on mutual trust, a servant and transformational leadership style, a shared cognitive perspective of hope and optimism, and fiscal transparency. In addition, Förster and Füreder (2021) emphasize resilience mechanisms of leaders and analyze how they contribute to the resilience of hospitals during the pandemic. They identified four key action areas: solving of structural problems, network(ing), anticipation and an open mindset, as well as individual resilience strategies. Concerning the latter, they particularly emphasize individual resilience strategies (that include physical and emotional aspects) for coping with the pandemic. The authors also highlight networking within the hospital as essential and consequently horizontal interactional resilience mechanisms. Finally, the conceptional work by Mithani (2020) provides multilevel insights into resilience in life-threatening events (e.g., natural disasters). He distinguishes five resilience modes: avoidance (in terms of evading the threat), absorption (i.e., absorbing the devastating impact), elasticity (in terms of cognitive and physical flexibility), learning (development of new capabilities, skills etc.) and rejuvenation (i.e., redevelopment after complete desolation). This scholar also assigns (individual and organizational) resilience mechanisms to these five modes and differentiates between static and dynamic resilience.

In sum, our literature review shows that there is only a small body of research dealing with NPO resilience mechanisms during the pandemic (or in other disruptive extreme contexts) and that the identified (mainly qualitative) papers conceptualize resilience mechanisms heterogeneously. Those are sometimes considered to be a (rather unstructured) combination of capacities (management), processes and resources (see, e.g., Witmer and Mellinger, 2016; Hutton et al., 2021), or themes and tactics (cf., e.g., Searing et al., 2021) as well as characteristics, skills, abilities or competences (see e.g., Herberg and Torgersen, 2021). Besides, research predominately focusses on organizational level resilience mechanisms such as strategic planning, financial management or inter-organizational collaboration (Dayson et al., 2021; Hutton et al., 2021; Searing et al., 2021; Kim et al., 2022). We identified only a few empirical papers focusing on individual and relational resilience mechanisms of NPOs in extreme contexts (Witmer and Mellinger, 2016; Förster and Füreder, 2021; Herberg and Torgersen, 2021). These studies are limited, though, inasmuch as the findings of Herberg and Torgersen (2021) are limited to hierarchical (profit) organizations (e.g., military, private security) and thus need to be transferred to other types of organizations. Only Förster and Füreder's (2021) research focus is comparable to ours. Their findings, though, are limited to resilience of leaders and do not encompass interactional resilience mechanisms in general, while we focus on both individual-based and interactional resilience mechanisms.

Specifically, our paper aims to answer the research question, which individual-based and interactional resilience mechanisms helped Austrian social and healthcare service NPOs to cope with the COVID-19 pandemic as a disruptive extreme context. To answer this question, we conducted an exploratory study based on 33 semi-structured expert interviews with managers of 14 social and healthcare services NPOs in Austria.

## Materials and methods

### Research approach

As mentioned above, data was collected through semi-structured qualitative interviews. This kind of problem-focused interviews allows gathering detailed information and perceptions about specific circumstances from experts (Gläser and Laudel, 2009). In general, qualitative interviews seemed to be appropriate for our study due to their flexibility and their information-rich illustration of the phenomenon of interest (Patton, 2002). For exploring how social and healthcare service NPOs (can) succeed in coping with the COVID-19 pandemic a qualitative research design was chosen, as this enables in-depth evaluation of information given within the interviews. The qualitative paradigm primarily aims at an understanding-interpretative reconstruction of social phenomena in their respective context (Döring and Bortz, 2016). Data was analyzed using the qualitative content analysis (Mayring, 2015; Mayring and Fenzl, 2019). One of the main advantages of qualitative content analysis is its systematic nature, namely the rule-guided, step-by-step procedure according to a defined flow model.

### Recruitment and participants of the study

We decided to study NPOs of the social and healthcare sector as these organizations were crucial for coping with the pandemic, despite being severely affected by the pandemic themselves. In particular, we chose large social and healthcare service NPOs because they were key performers in political processes (e.g., they were consultants of the government) as well as in operative processes (e.g., they were responsible for testing, vaccinating, and caring for vulnerable individuals). We also focused on large NPOs due to our research interest in analyzing their formal crisis management, a “feature” which small NPOs are unlikely to have. In order to get information-rich illustrations and thus maximize the chances of observing our phenomenon of interest (i.e., resilience mechanisms), we relied on a purposeful sampling strategy, which allowed us to select participants that are well-informed about the phenomenon (Patton, 2002). We used a homogenous purposive sampling strategy, which focuses on choosing similar members (Patton, 2002, p. 235). Purposive sampling was based on formalized classification. We used the following two selection criteria:

- Austrian NPOs active in social and healthcare according to the registers of the lobbying or umbrella organizations “Interessenvertretung Sozialverband”, “Verband Sozialwirtschaft Österreich” and “Fundraising Verband Austria”.
- Large NPOs based on income thresholds: Organizations with revenues higher than three million euro or organizations with donations of more than one million euro (Vereinsgesetz, 2002).

To recruit appropriate participants for our study, we gathered e-mail or phone contact information of NPO managers *via* website research. Subsequently, we screened the potential participants for being either strategically or operatively involved in pandemic management. Moreover, we checked whether they had staff management responsibility. For testing and improving the semi-structured interview guide, two pilot interviews were conducted prior to the start of the interviews.

The selected NPOs cover a wide range of social and welfare services, such as caring and supporting homeless, elderly persons, refugees or children within residential facilities, food delivery, family support, leisure activities, employment opportunities, and education. Their fields of activity also include healthcare services for physically or mentally disabled persons, people living in difficult psycho-social situations, as well as injured or sick persons, and thus involve the provision of, e.g., (psycho-) therapies, palliative care, ambulance services, mobile care, or blood donations.

**Figure 1:**
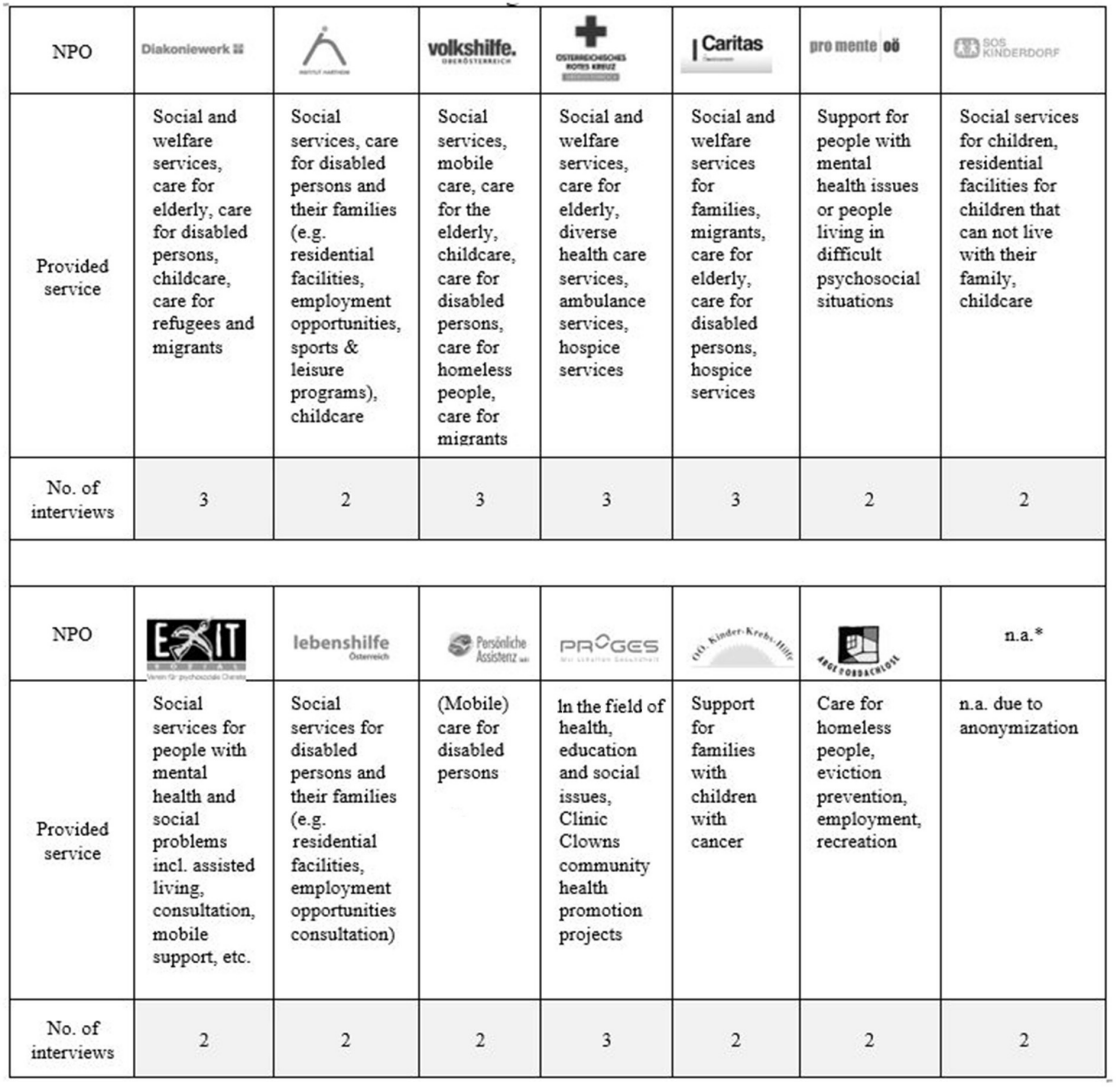
Sample of the study.

We interviewed top managers (i.e., CEOs and members of the board of directors) as well as mid-level managers (e.g., operating managers of a division or unit). We chose top and mid-level managers who were engaged in pandemic management, respectively, had corresponding decision competencies. The final sample includes 33 NPO managers (~ 60 % male and 40 % female managers) of 14 Austrian social and healthcare service NPOs. The detailed sample is presented in Figure 1.

We continued sampling and contacting suitable participants until we gained a satisfactory diversity of roles and functions of our interviewees and foremost, until the interviewees' content contributions were not fundamentally new, respectively, there was information redundancy. Consequently, we could not develop further codes based on additional interviews. We achieved thematic saturation (Patton, 2002) with interview 30. Because at that point, three more appointments were scheduled, we also conducted these additional interviews.

### Ethics of the research

At the beginning of the interviews, we briefed the interviewees on the aims, procedure, and publication plans of the results; this also included the issue of anonymization. Interviewees could choose to anonymize their name, job title and the name of the organization. Only one participant chose full anonymization (name, title and organization). All other participants only chose to anonymize their names. All participants gave verbal consent to videotaping and publishing the results of the interviews. The consent of using the data and anonymization was given during videotaping. Respondents were asked to let the interviewers know, if they had any questions or felt uneasy. Answering the questions was voluntary, the interviewees were able to skip questions or decline answering specific questions (which was not the case for any interview). The participants had the possibility to end the interview at any time and also to withdraw from the interview (respectively, the data analysis later on). Only the researchers had access to the data. No ethics committee was necessary as this is unusual for studies like ours in Austria.

### Quality criteria

We refer to quality criteria defined by Lincoln and Guba (1985) as this is one of the most cited criteria schema (Döring and Bortz, 2016). This includes the quality criteria of credibility, transferability, dependability and confirmability. Credibility means that the results and interpretation of data are trustworthy and, in the end, leads to internal validity of the study. Transferability means that the results and conclusions from the study are transferable to other contexts (in this case other extreme contexts). Transferability should lead to external validity. Dependability means that the research process is comprehensible and reproducible. This leads to reliability of the data and the study. Confirmability means that the study results should not be influenced by prejudices, interests or perspectives of the researchers. Confirmability should lead to objectivity of the study as well as to relevance and ethic rigor. In the study at hand the quality criteria are ensured and achieved by the following techniques (Lincoln and Guba, 1985; Döring and Bortz, 2016):

- comprehensive data collection through a long period of time in the field (see data collection);
- verification of the interpretations on the basis of the raw data;
- triangulation by a stepwise replication of data by the researchers (also intercoder reliability);
- debriefing of the study with outside peers (e.g., discussion on different conferences) and
- description of the studied organizations and contextual conditions in order to make transferability of the results to other organizations and contexts possible.

### Data collection

Due to legal COVID regulations, the semi-structured expert interviews were conducted virtually *via* Zoom. The interview team included the four authors. All interviews were done in pairs (meaning two researchers and the NPO manager). Interviews were conducted in the time span of 5 months (from October 2020 to February 2021). The interviews lasted between 20 and 94 min, with an average duration of 49 min.

The overall purpose of the interviews was to gain insights into the perceptions of the experiences, responses, and learnings of the pandemic from its beginning in March 2020 until February 2021. Therefore, a semi-structured interview guideline with a total of eight deductively derived open questions was developed to enable intersubjective comparability (Lamnek, 2010), but also to allow probing (additional) questions to obtain detailed insights and complementing information.

The interview guide was structured as follows: The first question addressed the interviewees' job description in the NPO, encompassing their routine as well as their non-routine job when coping with the pandemic. The second question dealt with the challenges the experts experienced during the crisis in general. This was followed by question three which specified the pandemic challenges by asking, whether and to what extent managers were confronted with challenges in the task, physical, social and temporal context. Number four referred to the crisis management of the NPO—its nature and decision-making/implementation. Question five raised the question, whether cooperations were useful for coping with the pandemic (and if yes, which collaborations with whom). Question six aimed at identifying the most important aspects the managers learned from the pandemic. This question related to individual, relational and structural/organizational factors facilitating organizational resilience. Question seven explored whether the organizations prepare for similar crises (and if yes, how they prepare). Finally, in question eight, the interviewees were encouraged to declare which resources they would need for better coping with such an exceptional situation. Data was gathered by screening the whole interviews, whereas the most information with respect to the context and to resilience could be generated from questions two and six.

### Data analysis

In a first step, we prepared verbatim transcripts of the videotapes for data analysis. We decided to apply qualitative content analysis according to Mayring (2015) due to its flexibility regarding to the material and at the same time its predefined process schema. Following the flow model of Mayring (2015) we used both a deductive and an inductive approach for defining categories.

The data was coded by two authors. In a first step, each author individually coded the transcripts in order to create an initial coding schema. Team reflexive dialogue and reflexive writing helped us to reflect, critique and assess subjectivity and the context as research influencing factors (Olmos-Vega et al., 2022). To ensure intercoder reliability, two authors met to discuss the codes (refine, adapt, and integrate new ones) at least once a week. Thus, in an iterative process, we added and revised our coding schema and the paraphrases. This also included a recheck of the paraphrases with regard to consistency and meaning. Following the model of Mayring (2007), we paraphrased, generalized and reduced the text passages and created main and subcategories. This guarantees to meet the quality criterion of a systematic, rule-bound procedure (Mayring, 2007).

Data analysis focused in a first step on the challenges for NPOs due to the pandemic. We used inductive category formation to identify the pandemic challenges. For creating corresponding categories based on the interview data, we selected all text passages in which the participants mentioned any difficulties, non-routine tasks or tasks modified due to the pandemic. As a result, main categories are hence defined as business and leadership-related challenges.

In a second step, data was inductively analyzed to explore individual-based resilience mechanisms. Thus, we selected all text passages where participants indicated any individual resources which facilitated organizational resilience. As a result, main categories were defined as attitudes and personality traits. Relying on the understanding of interactional resilience mechanisms as non-specialized social resources, we searched data also for text passages associated with social capital. Based on the social capital classification of Nahapiet and Ghoshal (1997), we developed a deductive coding schema consisting of category definitions, anchor examples and coding rules (see Table 1) (Mayring, 2015). During our text analysis, additional inductive sub-categories complemented the deductive coding schema.

**Table 1:** Coding schema for interactional resilience mechanisms (own elaboration).

**Category and title**		**Definition**	**Anchor example**	**Coding rule**
Relational social capital	1.a Social connectedness as team (network) capital.	Resources created and leveraged from horizontal relationships between individuals at the same hierarchical level (cf. Badura et al., 2013).	“Colleagues you can rely on each other are very important” (IP 4).	Only categorize, if the text passage is related to resources, which are embedded in or result from colleagues, staff members as “team” (network), respectively, from its corresponding interactions.
	1.b Managerial staff orientation as leadership capital.	Resources created and leveraged from vertical relationships between staff and leaders (cf. Badura et al., 2013).	“You cannot express your gratitude, your respect and appreciation often enough” (IP 17).	Only categorize, if the text passage is related to resources, which are embedded in or result from the relationship between the leader and the staff, respectively, from their corresponding interactions.
	2. Shared crisis understanding as cognitive social capital.	“Resources, which represent shared understanding, interpretations and systems of meanings between parties” (Nahapiet and Ghoshal, 1997, p. 35).	“There is much vigor […] vigor which results from the common past” (IP 11).	Only categorize, if the text passage is related to values, norms, beliefs and meanings which are shared from the organizational members—commonly practiced in everyday life and are considered to be obligatory.

## Results

In advance of presenting the individual-based and interactional resilience mechanisms, we start with a brief illustration of the challenges experienced by the NPO managers in the pandemic.

### Challenges of social and healthcare service NPOs in the COVID-19 pandemic

**Figure 2:**
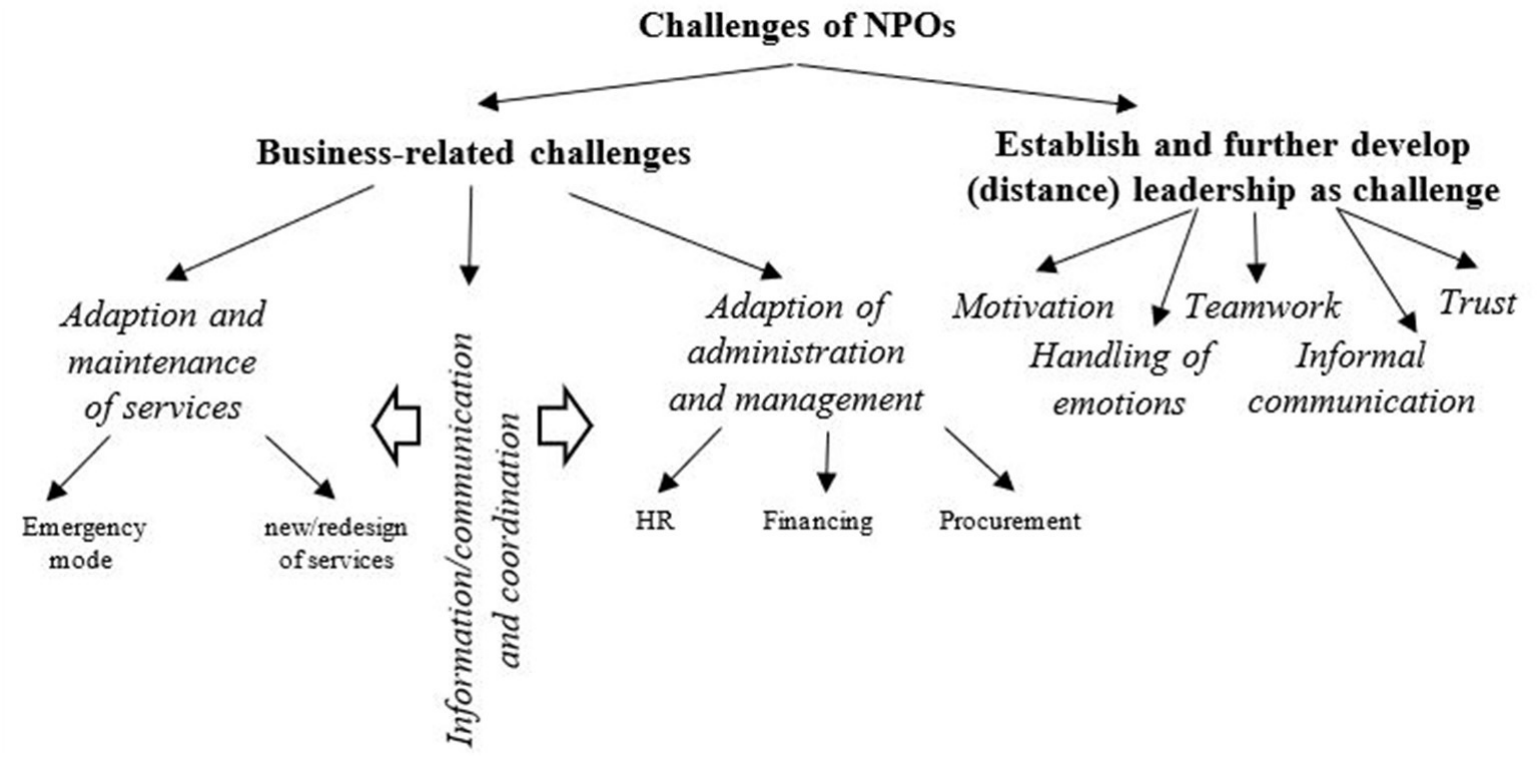
Challenges of social and healthcare services NPOs in the COVID-19 pandemic.

Due to far-reaching governmental regulations, there were various decisive business-related challenges to cope with (see Figure 2). Business-related challenges refer to two domains: firstly, how to maintain and adapt the delivery of services and secondly, how to adapt administration and management. The first domain includes the establishment of an emergency operation mode and a new respectively (re-)design of services. In detail, interpreting governmental regulations (“in the beginning there were new regulations every day […]” IP 21) and also *ad-hoc* problem-solving (“there was a strong need for adhoc response but no unnecessary reflexive reactions” IP 5) challenged the NPOs in establishing an emergency operation mode. With respect to services, NPOs struggled with the question which services represented core services and thus had to be provided necessarily and which services were not such ones. NPOs also had to decide which core services should be provided as in-person operations which in turn implied to apply hygienic protective measures. In this context, NPOs sometimes faced a dilemma: “We had to adhere to hygienic protective measures. At the same time, we were asked not to be scared to death and act courageously” (IP 6). Likewise, NPOs had to develop new online services for clients or re-design existing services as online services. This often resulted in a modification of the methodical, therapeutical or didactical approach of the services.

The adaption of administration and management includes, primarily, adjusting human resource management, financing, and procurement. We identified the following corresponding challenges:

- human resource management: developing recruiting, onboarding and training in virtual contexts, coping with dynamic manpower requirements including staff shortages due to illnesses and care, establishing shift work, enacting short-time work and the corresponding payroll accounting;
- financing: finding alternative sources of funding due to losses of revenues, applying for the “NPO fund”^1^;
- procurement: providing technical equipment in terms of IT hardware (e.g., laptops, webcams or headsets) as well as personal protection equipment.

In line with the adapted service delivery, administration and management NPOs also had to modify information, communication and coordination processes, as illustrated in the following quote: “So, there was a lot of reorganizing at the organizational level” (IP 15). With regard to information and communication, NPOs struggled with gathering reliable expertise and information. Moreover, it was a challenge to guarantee short, clear and understandable crisis communication for the different target groups (not only staff, but also clients and their families), as one participant stated: “Of course, it was also a great challenge to inform and instruct the employees” (IP 9). Main challenges referring to coordination were aligning formal crisis management to permanent management as well as implementing various (bilateral) mutual coordination tasks.

In addition to business-related challenges, the second core challenge was to establish and further develop (distance) leadership. This primarily included considerations of how to motivate and integrate remote staff, because after the first weeks of home office, it became evident that there was a need for cultivating teamwork and enhancing informal communication. Moreover, leaders had to think about how to maintain and foster trust *via* distance. Finally, leaders also faced an enhanced spectrum of emotions and mental health issues of staff, clients, and partners, such as uncertainty, panic, frustrations or over-motivation as the following quote illustrates:

“Employee reactions were split (…) between the positions of ‘that is all not so bad', ‘that is grossly exaggerated', to the point of mortal fear. I had employees in fear of death who were no longer able to work at all; in middle and upper management, too. That was a big problem, because when these people are absent, I can't say, ‘stay at home and stay safe', because business has to go on” (IP 12).

Thus, dealing with emotions evolved into a leadership task of increased relevance.

### Resilience mechanisms

**Figure 3:**
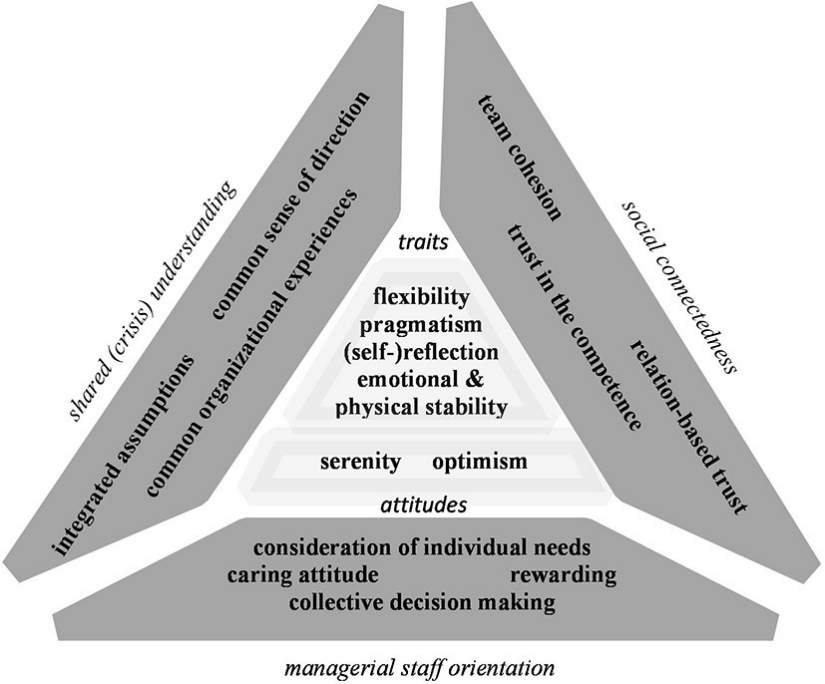
Framework of individual-based and interactional resilience mechanisms (own elaboration).

Based on the interview data, we developed Figure 3, which provides an illustration of the identified resilience mechanisms of NPOs in the COVID-19 pandemic. As mentioned before, we focus on individual-based and interactional resilience mechanisms. Individual-based resilience mechanisms as nucleus of organizational resilience represent the core of the figure. We identified two main categories of individual-based resilience mechanisms. These are personality traits and attitudes. The individual-based mechanisms are surrounded by the triangle shaping interactional resilience mechanisms whereby the triangle is considered to symbolize unity and ascending force. Each side of the triangle refers to a main category of interactional resilience mechanisms. These are a shared (crisis) understanding, social connectedness and managerial staff orientation.

#### Individual-based resilience mechanisms

Data show that the subcategories serenity and optimism as attitudes and the subcategories (self-)reflection, pragmatism, flexibility and individual stability (both emotional and physical) as personality traits constitute the individual-based resilience mechanisms. These individual-based resilience mechanisms refer to both managers and general staff.

##### Individual-attitudes-as-resilience-mechanisms

The COVID-19 pandemic highlights that ***serenity*** was an important resilience mechanism, as one interviewee describes: “Stay cool, we will get through it in the end” (IP 8). Serenity prevents narrowed cognitions that are common in panic situations. Staying aware of signals and sensing different alternatives for action certainly contributes to the capacity to act. Two interviewees coined the term “serious serenity” (IP 4, 11), which specifically suggests that serenity corresponds to a well-founded rather than a reckless coping with challenges. This also includes being mindful and attentive toward colleagues, staff, and clients. With regard to the new COVID-19 regulations, this meant to carefully implement the rules without neglecting the specific needs of the clients.

Further, a ***sense of optimism*** was mentioned as resilience mechanism, as the following quote illustrates: “Optimism plays an important role. I think that optimism provides a basis for dealing with changes, which are not positive at first sight” (IP 11). Thus, optimism helps to cope with unpleasant situations or disturbances. When no escape is possible, warming toward the situation facilitates problem-solving. Interviewees also positively associated optimism with self-efficacy. Moreover, interviewees emphasized that optimism strengthened job motivation. In particular, optimism coupled with a sense of humor created “team spirit” and (a sense of) togetherness.

##### Personality-traits-as-resilience-mechanisms

We also see that a sound ***pragmatism*** in decision-making, in terms of having the courage to leave a gap in decision-making, was crucial for coping with the pandemic, because there often was an incomplete and/or partly inconsistent information base. Moreover, it is essential because extreme situations limit an organization in planning or making forecasts, as the following quote shows: “Coping with a crisis always includes that some questions remain unanswered; it is not possible to clarify everything” (IP 4). Pragmatism in decision-making certainly contributed to an enhanced orientation toward solutions and especially to quick solution finding, which was important due to the dynamics and uncertainty of the disruptive extreme context. Quick solutions in turn represented the basis for *ad-hoc* organizing.

Moreover, individual ***flexibility*** is considered to be an indispensable mechanism in fighting the pandemic. Flexibility refers to cognitive as well as to spatial or temporal flexibility. Cognitive flexibility manifested in an elastic mindset, as the following quote demonstrates: “To be flexible often means to think differently, to integrate the new circumstances in your thinking” (IP 23). Cognitive flexibility also referred to coordination processes, e.g., when to choose which coordination processes, as one interviewee describes: “It often happened this way. [you had to decide] when you need a crisis committee, or when bilateral agreements in the team are adequate” (IP 6). Moreover, physical flexibility in terms of switching between office, home office or different organizational units, and temporal flexibility of individuals in terms of flexible working hours, e.g., in the evening, on weekends, or shift work, supported organizational functioning and adaptation.

A further common aspect across interviews is that ***(self-)reflection*** was crucial for successfully coping with the situation. Self-reflection is particularly important in chaotic situations with a lack of control from outside or of institutionalized norms and rules that individuals usually rely on. Self-reflection referred to aspects of daily-business life, as the next quote exemplifies: “[In online meetings] it is necessary to reflect in advance which information you need from whom or which information could others require. What is absolutely necessary to clarify?” (IP 24). Self-reflection also included reflecting on how to achieve a new form of work-life balance under these changed circumstances, as one interviewee illustrates:

“You should not wear a jogging suit all the time because you are in home office. It is necessary to get dressed up sometimes […]. Well, [in sum] it is important to prevent sloppiness” (IP 24).

With regard to leaders, it was necessary to reflect one's role or position as leaders, particularly due to new or altered needs and requirements of staff and peers.

When facing a disruptive extreme context, ***individual stability*** also plays a significant role. Individual stability refers to physical as well as to emotional fitness in terms of being emotionally and physically persevering and durable. Individual stability can be considered crucial, because there was an enormous workload across daily work tasks as well as there were many social and emotional challenges, in particular at the beginning of the pandemic (prevailing the first lockdown in March 2020). Potentially combined with personal dismay (e.g., COVID-19 infections of family members), this mostly represented a heavy burden for leaders and staff members, as one interviewee stated: “The COVID-19 pandemic represents a liminal experience, in particular referring health” (IP 14). Thus, individual stability is the foundation for individual resilience. Interviewees also stressed the importance of conscious breaks and hours to relax, e.g., pets or hobbies, such as jogging or fly fishing, for maintaining individual stability. In particular, with regard to emotional stability, they emphasized the importance of discussing emotional constraints in teams, whilst also considering the self-responsibility of individuals a crucial pillar.

#### Interactional resilience mechanisms

As illustrated above, a shared (crisis) understanding, social connectedness, and managerial staff orientation represent the main categories of interactional resilience mechanisms. A shared crisis understanding consists of the subcategories creating a common sense of direction regarding the new business normal; developing it into integrated assumptions and, finally, in exploiting common organizational experiences from the past. Social connectedness encompasses the subcategories team cohesion, relation-based trust and trust in the competences. Finally, managerial staff orientation is based on the following subcategories: consideration of the individual characteristics and needs of the staff, a caring attitude, an emphasis on appraisal, and collective decision-making.

##### Shared-(crisis)-understanding-as-cognitive-social-capital

Having a shared understanding (of the crisis) refers to common beliefs, interpretations and meanings (Nahapiet and Ghoshal, 1997). Subsequently, we describe the identified subdimensions (common sense of direction regarding the new business normal; integrated assumptions, and common organizational experiences).

A ***common sense of direction*** of organizational members represented a crucial resilience mechanism. Particularly at the beginning of the pandemic, there was an overwhelming sense of insecurity and disorientation with regard to the handling of the virus but also, whether and how daily business might run. Thus, achieving a common sense of direction referring to a shared understanding about the new daily business including COVID-19 regulations represented a central asset for being productive, as the following quote exemplifies:

“No one can answer [how operational processes will be in the next month], that is why, we don't do forecasts, but we would like to convey the impression and I think we succeeded in it… that we a prepared for all eventualities” (IP 8).

The sense of security also included that staff was provided with contacts in case of emergency, e.g., COVID-19 infection. The sense of security, in turn, enhanced the mood and motivation of the staff. This common sense of direction represented the most elementary form of mission orientation.

In the course of the pandemic, this first common sense of direction regarding daily business often evolved into corresponding ***integrated assumptions***. Integrated assumptions relate to a shared understanding, how business might be run under conditions of the pandemic at least in the medium term, referring to shared agreements about, e.g., home office rules or human resource policy (e.g., handling of short-time work). Integrated assumptions also refer to how to realistically apply protection rules, as the following quote illustrates: “The legal regulations [...] sometimes were absurd and not implementable. We discussed a lot […] until we found an agreement how to deal with the regulations in the organization” (IP 28). Integrated assumptions represented a crucial prerequisite for the organizations to remain able to act. Additionally, integrated assumptions also referred to a shared understanding of how to cope with failures. This was very important because one cannot prevent failures in crisis. Failure tolerance of staff took pressure away from both leaders and staff. Moreover, it enhanced personal initiatives of staff.

Moreover, ***common organizational experiences*** which relate to experiences that staff have gained in their current organization (Barrett, 2018) represented an often-mentioned resilience mechanism. Common organizational experiences refer to both experience in teamwork and in various cooperation activities within the NPO. With regard to social and healthcare service NPOs active in crisis management, it also refers to common organizational experience in coping with crises, e.g., natural disasters, psychosocial or financial crises, as illustrated in the following quote: “The focus is now on crisis management but we are familiar with it; we are trained. We will succeed. That is not a problem at all” (IP 10). Moreover, interviewees also mentioned that the common experience about the first lockdown was helpful for the following lockdowns or more general running business under COVID-19 restrictions. One interviewee describes this aspect as follows:

“I would like to emphasize that the longer the pandemic lasts, the more experienced, cooler and judicious we are […]. Compared to spring [first lockdown], we have gained experiences regarding online team meetings, crisis communication, online team building” (IP 28).

From the interviewees' perspective, the organizations which are generally active in crisis management were less paralyzed by the status of the pandemic, because crises of different natures are their business. Experiences in teamwork or collaboration facilitated coping with the pandemic, because this was associated with informal knowledge, relation-based trust as well as with the acceptance of top-down decisions and in general with vigor. This is well-exemplified in the following quote: “There is much vigor, much power for dealing with this situation… much vigor results from the common past” (IP 11).

##### Social-connectedness-as-team-(network)-capital

In addition to a shared crisis understanding, we identified social connectedness as a further important resilience mechanism. Social connectedness relates to a sense of belonging to colleagues (Stavrova and Luhmann, 2016). Social connectedness consists of three pillars. These are team cohesion, relation-based trust as well as trust in the competences.

***Team cohesion*** in terms of “an engagement in and commitment to a group” (Bowers et al., 2017, p. 9) is also considered to be beneficial for coping with an extreme context, as illustrated in the following quote: “What was pleasant was the team cohesion, to pull together, to collaborate, to find common arrangements […] without rush jobs of anyone, but always in agreement” (IP 17). Team cohesion manifested in a multifaceted engagement of staff, a high commitment to work as well as in taking care of each other. Leaders were even proud of the team cohesion. In turn, the pandemic also enhanced team cohesion, as one interviewee stated: “This catastrophe, this common [experience], coping with this situation has connected us one to another” (IP 11).

Moreover, ***relationship-based trust*** was highlighted as a resilience mechanism—it was even regarded as “indispensable” for coping with the pandemic (IP 4). Relationship-based trust refers to mutual trust in terms of relying on each other. This includes trust between team members, organizational members, leaders, and network partners. Relation-based trust represented the basis for information sharing and communicating, and thus affected “crisis” response seminally. Relationship-based trust also fundamentally improved collaborative effort, because the feeling of relying on each other provided a sense of security: “Relying on each other […] day and night, on the weekend… I think that is really the right approach” (IP 11). Furthermore, trust helped to substitute formal structures and procedures, as suggested by the following statement: “Having mutual trust is essential for coping with the crisis. Organizational structures won't accomplish what trust accomplishes” (IP 23). Comparable to the positive feeling of team cohesion, leaders were also proud to feel the trust of their staff (IP 22).

In addition to relation-based trust, having ***trust in the competences*** of the staff and of the leader themselves also boosted resilience. Trust in the staff's and leader's competences actually resulted in faster decision-making processes and it represented the base for autonomous action, creativity and improvisation. These effects are well-demonstrated in the next quotes:

“[I became aware] that there are many good [i.e., competent] employees who you can trust … [employees] who can handle responsibility” (IP 19).

“There was much improvisation [done by the employees], they did a lot without my supervision or my commands… I am very impressed. I have always supposed that I can trust my employees, they will make it… but I had never thought that they would make it in such an overwhelming (positive) extent” (IP 12).

It is worth mentioning that the effect between trust in competences and the pandemic was not a one-way relationship. Rather, the pandemic also improved the trust in the competences of the staff, the leaders and the organization.

##### Managerial-staff-orientation-as-leadership-capital

Whereas social connectedness and its subdimensions emphasize the vigorous resources emanating from teams or collaborations without considering any hierarchies, managerial staff orientation refers to resilience mechanisms created and leveraged from vertical relationships between staff and leaders (cf. Badura et al., 2013). Managerial staff orientation encompasses various subdimensions. Firstly, it refers to a manager's effort to consider the individual characteristics and needs of the staff, secondly to a more caring attitude compared to “daily business”, thirdly to an enhanced emphasis on appraisal, and finally to collective decision-making.

Before discussing it in detail, we would like to stress that interviewees highlighted that the physical presence of the managers in office was crucial for dealing with the COVID-19 pandemic. According to the interviewees' experience physical presence was associated with a better (informal) knowledge sharing, an enhanced involvement with challenges, and had a symbolic effect in terms of “fighting side by side”. Some staff members even interpreted a lack of physical presence as loss of trust.

Interviewees stated that the pandemic intensified the manifestation of various positive or negative characteristics in individuals, e.g., the egoists became even more egoistic, and the loyal ones became even more faithful and cooperative. Thus, it was necessary to ***consider*** the corresponding increased ***individualized needs***, e.g., regarding communication, feedback, and supervision, in order to provide the smoothest operations possible. The following quote regarding home office refers to this individualization:

“I assumed that home office would fit well to everyone, but I experienced that the home office was a heavy burden for some staff members, for others it fit surprisingly well. Both are legitimate” (IP 23).

Moreover, staff orientation manifested in a more ***caring attitude*** compared to “daily business” because the pandemic involved a broad spectrum of (intensified) emotions, such as panic, fear, shock, insecurity, frustration, nervousness, irritations, and loneliness, as mentioned above. Thus, an interviewee stated: “As leaders we were more challenged, in particular regarding [staff] motivations and emotions” (IP 15). Through intense caring in terms of attentively perceiving and regulating emotions, managers were able to enhance motivation and performance. Caring also included looking after staff with regard to overworking and burnout, e.g., by limiting extra working hours or enabling brief timeouts.

There was also evidence for the importance of ***appraisal*** for employees who preserved and performed outstandingly, although many of them were simultaneously challenged in their private lives (e.g., homeschooling, caring for infected persons). Most interviewees stressed that it was essential to reward the loyalty of staff. A correspondingly active rewarding can be of tangible or intangible nature. “One can't say it often enough—either writing ‘thank you' in a letter or saying ‘thank you' face-to-face, expressing gratitude and valuation” (IP 17).

Finally, managerial staff orientation refers to collective decision-making. Where possible, ***collective decision-making*** seemed useful because it contributed to motivation and reduced resistance. This aspect is well-illustrated in the following quote:

“It is very important to get the staff on board… I can take the lead, I can swim ahead, but if no one joins me in swimming, I won't accomplish anything” (IP 17).

In summary, the COVID-19 pandemic changed managerial staff orientation, as the following quote exemplifies: “You have to switch your mode; do not believe that you can continue leading as usual. It won't work. A different form of leadership is needed; in particular I have to assist my colleagues more immediately” (IP 5).

## Discussion and contributions

Our findings show numerous resilience mechanisms suitable for facing disruptive extreme contexts. Whereas most current research about NPOs dealing with the pandemic or disruptive extreme contexts focuses on resilience mechanisms at organizational level, we researched individual-based and interactional ones.

Our study reveals that interactional resilience mechanisms essentially contributed to generate a basis for facing the adversity. It required the collective competence, the collective spirit—in sum, sound relations to overcome the uncertainty and the diverse obstacles. Thus, Hannah et al. (2009) attributes social resources to attenuate the effects of adversity crucially. It seems that these mechanisms evoked a certain sense of security and enhanced empowerment of staff. This is in line with Williams et al. (2017) who emphasize that relations are the bedrock for activating cognitive, emotional and behavioral abilities. In particular, trust played an important role because it enabled autonomous action. Thus, staff members could suggest their new ideas and solutions to colleagues and implement them. Also, team cohesion as further interactional resilience mechanism certainly had a positive effect. Team members felt “pulled together” and partly also obliged to support each other. This in turn enhanced team effectivity. Adler and Kwon (2002) refer to this effect as social solidarity. Our results also show that the relationship between staff members and leaders has changed in the pandemic—it intensified and became more active. Therefore, we regard managerial staff orientation as a crucial lever for facing an extreme context. The corresponding strengthened consideration of individual characteristics, the enhanced caring attitude, the emphasis on appraisal, and finally the integration of staff in decision-making helped to reduce social, emotional and health hardships. Similarly, Witmer and Mellinger (2016) highlight that an attitude of managers which evokes a feeling of being supported, is essential. Collective decision-making was also essential. On the one hand it enhanced cohesion, on the other hand thus, staff members contributed their knowledge to solve problems, as Hannah et al. (2009) describe. Overall, we would like to emphasize that the collective effort necessary for coping with the disruptive extreme context has a vertical as well as a horizontal perspective. Nonprofit managers contributed vertically *via* managerial staff orientation as well as staff members contributed horizontally *via* social connectedness and common sense of direction. This mix of vertical and horizontal efforts was necessary, because only vertical efforts were not efficient enough for the surprising and hectic situation (Van der Vegt et al., 2015).

Furthermore, it became evident, that individual-based resilience mechanisms fundamentally affected organizational resilience. Individual traits and attitudes were beneficial in various ways. Self-reflection and flexibility facilitated individuals to get the most out of themselves. In other terms, these mechanisms had a (self)-activating effect and kept individuals going, respectively problem-solving. They made “hidden capabilities” salient, as Hällgren et al. (2018) propose. According to expert statements this resulted in lots of creative and innovative solutions. Thus, similar to existing studies our study also reinforces that individuals enhance the amount, access and quality of resources for deploying and recombing resources in new ways (Sutcliffe and Vogus, 2003). Our results also confirm that in particular, flexibility and pragmatisms supported *ad-hoc* organizing (Williams et al., 2017). They helped to substitute inadequate or missing processes and systems, e.g., decision-making processes and information systems and thus guaranteed acting. Correspondingly (interacting), individuals configured resilience by filling the gaps resulting from the disruptions of the extreme context. This is also stressed by Majchrzak et al. (2007) who point out that acting is essential for facing adversity; from time to time even more than rules.

As mentioned above, extreme contexts raise lots of (positive and negative) emotions. Particularly during the first months of the pandemic, emotions and mental issues were all-pervasive and hence represented an important issue to deal with, in order to restore or maintain the individual mental wellbeing. Due to the fact that emotions shape cognitions and behavior as well as relations, the regulation of emotions also became crucial from an organizational point of view. Several interviewees told us that in particular, negative emotions, such as fear or panic impeded coordination and service delivery. Thus, our findings also provide evidence that coping with emotions is a significant resilience mechanism. This refers to the perception and handling of emotions within an individual as well as between individuals. This is in line with Herberg and Torgersen (2021) who emphasize emotion efficacy as crucial competence for coping with extreme events. In this regard, emotional efficacy refers to the competence, “how effectively a person […] experiences, exploits, and responds to a full range of emotions in a contextually adaption and valued-based manner” (p. 18). Jalil et al. (2021) also emphasize that emotional coping strategies represent a crucial pillar of organizational resilience next to problem-focused coping mechanisms.

This study makes the following theoretical contributions to the field: Foremost, the study contributes to extreme context research. By identifying resilience mechanisms (at individual and interactional level), we illustrate non-specific coping mechanisms. Disruptive extreme contexts require such a non-specificity, because they have a surprising and unforeseen nature and therefore impede specific preparations. Thus, we extend knowledge about mechanisms that are “suitable” for coping with disruptive extreme contexts, for which only rudimentary research exists so far. Accordingly, we provide evidence that a fusion of extreme context research and resilience research essentially contributes to the understanding of how to cope with a disruptive extreme context.

In addition to extreme context research, we also contribute to NPO resilience research. By illuminating a broad range of individual-based and interactional resilience mechanisms, we complement the prevailing organizational-level NPO resilience research; we open the “black box” of resilience at organizational level. Thus, we offer alternative explanations as to what constitutes a resilient social and healthcare service organization. By identifying individual-based and interactional resilience mechanisms we provide deeper insights into NPO resilience. Whereas previous NPO resilience studies mainly refer to interactional resilience mechanisms with “externals”, e.g., with the community or partners, we focus on internal interactional resilience mechanisms (see Figure 2). In this context, we would like to stress our findings regarding managerial staff orientation as a particular research contribution. Managerial staff orientation, which includes the managers' efforts to consider the individualized characteristics and needs of the staff, a more caring attitude, an enhanced emphasis on appraisal, and collective decision-making, illustrates leadership capital as one dimension of social capital without focusing on a specific leadership style or competence.

Thirdly, we also nuance previous resilience studies. In this context, we particularly refer to Mithani (2020) and Herberg and Torgersen (2021). With our focus on social and healthcare service NPOs and thus predominantly non-hierarchical organizations, we partly complement the findings of Herberg and Torgersen (2021) who focus in their research on hierarchical organizations, e.g., the military. Concerning individual level mechanisms, scholars highlight trust, integrity and empathy as important attitudes. We particularly highlight the role of serenity and optimism. Moreover, we enrich their individual level competences by adding pragmatism as a crucial mechanism. Concerning interactional mechanisms, we found evidence for an intensified importance of a shared crisis understanding compared to Herberg and Torgersen (2021). This may be rooted in the fact that social and healthcare service providers as non-hierarchical organizations are comparably less familiar with situations assessments as, e.g., the military or police are. Moreover, generating a shared crisis understanding presumably is more important in disruptive extreme contexts than in Herberg and Torgersen's (2021) unforeseen contexts in general, encompassing e.g., also emergency contexts. We also nuance the findings of Mithani (2020). He obviously provides an excellent range of individual and organizational resilience mechanisms. Our findings provide evidence, though, that interactional (relational or group) resilience mechanisms should be defined as an own category or level (see also Witmer and Mellinger, 2016; Williams et al., 2017). On the one hand this underpins the fact that various interactional mechanisms are embedded in and generated from relationships, and on the other hand, this emphasizes the collective effort nature of resilience.

Fourth, it seems reasonable to propose that an identification of resilience mechanisms from a social capital perspective is appropriate and valuable, because it provides a consistent and clear framework for classifying resilience mechanisms at least at the level of a mid-range theory. This can improve the partly unclear and confusing (synonymous) use of competences, skills, abilities, qualities etc. for conceptualizing resilience mechanisms.

Concerning contributions to practice our findings clearly indicate that NPO managers who are responsible for resilience should concentrate on emotional and physical stability (of staff and themselves) as fountainhead for bearing exhausting periods. This includes preventive health and social care initiatives as well as adequate working conditions in daily business in advance of a “crisis”. Moreover, our findings call on organizations to intensively deal with emotions. Because extreme contexts fuel emotions and cognitions as well as behavior are inseparable from emotions, leadership trainings should focus on the handling and regulation of emotions. Finally, dealing with disruptive extreme contexts requires that both managers and employees get more familiar with such contexts. This proposes “crisis simulations” and trainings comparable to high-reliability organizations as well as developing a culture, which nurtures learning, creativity, fault tolerance, and flexibility. In sum, this enables NPOs to an emotion-focused as well as problem-focused coping with adversities (c.f. Jalil et al., 2021).

## Conclusion, limitations and further research

We aimed at identifying individual-level based and interactional resilience mechanisms, which fostered social and healthcare service NPOs in maintaining and adapting their functioning during COVID-19 pandemic. Figure 3 provides an overview of the identified resilience mechanisms. Firstly, our findings underscore that there is no “one best way” for coping with a disruptive extreme context in terms of e.g., an emergency plan, but there is a beneficial approach, which consists in continuously focusing on NPO resilience and its mechanisms. Secondly, the evolving nature of resilience became clear. Common organizational experiences, team cohesion, trust etc. do not arise overnight; their development takes time. The evolving character also manifests “during the crisis”, where interacting individuals make hidden capabilities salient. Finally, it also includes integrating the learnings for the next adversity in the “post crisis phase”. Overall, cultivating “powerful” resilience requires a corresponding continuous consciousness as well as an adequate (financial) resource allocation. We would like to conclude with an analogy of one of our interviewees who compared a resilient organization with a house of cards.

“A house of cards is vulnerable to break down, when the wind comes sideways; it is stable, when the wind comes from the front. In the pandemic, the wind came from the front and we resisted [the adversities]” (IP 5).

The wind came from the front, because this NPO could rely on the appropriate resilience mechanisms.

Our findings must be viewed in the light of some limitations, though. The focus on large social and healthcare service NPOs may imply a limited transferability to small NPOs or (grassroot) initiatives active in other fields of activity, such as e.g., small environmental NPOs. Secondly, we did the interviews at the beginning period of the pandemic that means our findings are limited to the pandemic's manifestations and the corresponding response in that period. Thirdly, there are further limitations inherent to qualitative interviews (Althubaiti, 2016; Creswell and Creswell, 2018). This includes a potential bias resulting from our point of view, the researchers' perspective. A possible subjectivity of the researchers may negatively influence data analysis and interpretation and thus create researcher bias. Moreover, interviews represent retrospective and self-reported data which may be associated with social desirability bias and recall bias. The interview setting (e.g., time pressure of some leaders) and the interview design (e.g., different intensities of questioning) can also be a source for bias. Fourth, we have neither analyzed the interrelations between the identified resilience mechanisms, nor how they are linked to practices.

Thus, further research might focus on NPOs of different sizes and fields of activity for complementing our findings, particularly for testing our resilience mechanisms. Moreover, there is need for multilevel research in terms of focusing on the relations between the various mechanisms for a better understanding of the nature of resilience. We also regard a deeper exploration of the resilience mechanisms against the background of concrete context factors, e.g., social challenges as useful in order to elaborate resilience mechanisms for different challenges.

## Data availability statement

The data that support the findings of this study are available from the corresponding author KAK, upon reasonable request.

## Author contributions

All authors listed have made a substantial, direct, and intellectual contribution to the work. KAK and SS share first authorship. BG and SM share senior authorship. All authors approved it for publication.

## Conflict of interest

The authors declare that the research was conducted in the absence of any commercial or financial relationships that could be construed as a potential conflict of interest.

## Publisher's note

All claims expressed in this article are solely those of the authors and do not necessarily represent those of their affiliated organizations, or those of the publisher, the editors and the reviewers. Any product that may be evaluated in this article, or claim that may be made by its manufacturer, is not guaranteed or endorsed by the publisher.

## Footnotes

^1^The “NPO fund” is a fixed-cost subsidy for NPOs established in 2020 by the Austrian federal government (cf. Bundesministerium für Kunst Kultur, öffentlicher Dienst und Sport, 2021).
